# Glutamine Availability Regulates the Development of Aging Mediated by mTOR Signaling and Autophagy

**DOI:** 10.3389/fphar.2022.924081

**Published:** 2022-07-04

**Authors:** Jiao Zhou, Honghan Chen, Jintao Du, Haoran Tai, Xiaojuan Han, Ning Huang, Xiaobo Wang, Hui Gong, Mingyao Yang, Hengyi Xiao

**Affiliations:** ^1^ Department of Geriatrics, State Key Laboratory of Biotherapy, West China Hospital, Sichuan University, Chengdu, China; ^2^ Department of Otorhinolaryngology Head and Neck Surgery, West China Hospital, Sichuan University, Chengdu, China; ^3^ Development and Regeneration Key Laboratory of Sichuan Province, Department of Anatomy and Histology and Embryology, Chengdu Medical College, Chengdu, China; ^4^ Institute of Animal Genetics and Breeding, Sichuan Agricultural University, Chengdu, China

**Keywords:** aging, glutamine, mTOR, autophagy, cellular senescence

## Abstract

Glutamine is a conditionally essential amino acid involved in energy production and redox homeostasis. Aging is commonly characterized by energy generation reduction and redox homeostasis dysfunction. Various aging-related diseases have been reported to be accompanied by glutamine exhaustion. Glutamine supplementation has been used as a nutritional therapy for patients and the elderly, although the mechanism by which glutamine availability affects aging remains elusive. Here, we show that chronic glutamine deprivation induces senescence in fibroblasts and aging in *Drosophila melanogaster*, while glutamine supplementation protects against oxidative stress-induced cellular senescence and rescues the D-galactose-prompted progeria phenotype in mice. Intriguingly, we found that long-term glutamine deprivation activates the Akt-mTOR pathway, together with the suppression of autolysosome function. However, the inhibition of the Akt-mTOR pathway effectively rescued the autophagy impairment and cellular senescence caused by glutamine deprivation. Collectively, our study demonstrates a novel interplay between glutamine availability and the aging process. Mechanistically, long-term glutamine deprivation could evoke mammalian target of rapamycin (mTOR) pathway activation and autophagy impairment. These findings provide new insights into the connection between glutamine availability and the aging process.

## Introduction

Aging is a deterioration process accompanied by physiological integrity deficiency, body function impairment, stress-resistance decline and mortality increase. The deterioration is the primary risk factor for various age-related diseases, such as atherosclerosis, amyotrophy and neurodegenerative diseases (Mitchell et al., 2015; Costantino et al., 2016; Wyss-Coray, 2016). Intracellular changes are involved in this process, including cellular genome stability, proteostasis, nutrient sensing and mitochondrial function (Lópezotín et al., 2013; Srivastava, 2017; Carroll and Korolchuk, 2018; Ou and Schumacher, 2018). Additionally, the accumulation of damaged organelles and long-lived proteins has been closely implicated in cellular senescence (Cadenas and Davies, 2000; Anding and Baehrecke, 2017; Dupont et al., 2017). Autophagy has been considered a crucial mechanism to clear damaged organelles and long-lived proteins (Anding and Baehrecke, 2017; Ravanan et al., 2017). Therefore, increasing attention has been focused on the relationship between autophagy and aging/senescence in recent years.

Glutamine is one of the most crucial amino acids for cell proliferation and survival (Salabei et al., 2015). It is the precursor of many physiological molecules, such as amino acids and nucleotides (Starr, 1975; Lacey and Wilmore, 1990). It is also involved in the synthesis of nicotinamide adenine dinucleotide phosphate (NADPH), glutathione (GSH) and ATP (adenosine triphosphate)to maintain redox homeostasis and supply energy (Altman et al., 2016; Xiao et al., 2016). Moreover, glutamine has been identified as one of the vital amino acids in the regulation of mTOR, thereby affecting a diverse array of intra- and extracellular signal responses (Jewell et al., 2015; Zhu et al., 2015; Bernfeld et al., 2018). Currently, glutamine has been shown to be an amino acid involved in the regulation of autophagy (Ke and Coffer, 2012; Tan et al., 2017), whereas the influence of glutamine on autophagy is inconsistent in previous studies (Sakiyama et al., 2009; Chen et al., 2014; Zhu et al., 2015).

Previous studies have suggested an inextricable link between glutamine metabolism and aging. For instance, glutamine levels are lower in the brains of Alzheimer’s disease (AD) patients, and glutamine supplementation (GS) can reduce inflammation-induced neuronal cell cycle activation, tau phosphorylation, and ATM (ataxia telangiectasia mutation)activation in an AD mouse model (Chen et al., 2012). On the one hand, the glutamine antagonist 6-diazo-5-oxo-L-norleucine (DON) induces a senescence-like phenotype in young umbilical vein endothelial cells (HUVECs) (Unterluggauer et al., 2008), while glutamine deprivation (GD) stimulates the expression of genes encoding senescence-associated secretory phenotype (SASP) (Shanware et al., 2014). On the other hand, GS (glutamine supplenmention) is beneficial for healthy animals and humans, such as weight maintenance and cognitive function improvement (Huang et al., 2017; Shang et al., 2020). Furthermore, GS has been reported to contribute to lifespan extension in ataxia telangiectasia mutant (ATM) mice (Chen et al., 2016). Despite these cues between glutamine availability and protection from aging, the molecular events involved remain unclear.

In this research, we pursued the implication of glutamine on the regulation of aging by manipulating glutamine availability from GD and GS both *in vitro* and *in vivo*. Our findings demonstrate that glutamine availability certainly affects the development of cellular senescence and aging, which is largely mediated by its repressive regulation of autophagy. We also found that prolonged GD was positively correlated with Akt-mTOR pathway activity. Overall, our study provides novel evidence that the regulation of glutamine availability is a prospective antiaging strategy.

## Materials and Methods

### Reagents and Antibodies

Rapamycin (R0395), bafilomycin A1 (B1793), LY294002 (L9908), L-glutamine (G3126), and D-galactose (G5388) were from Sigma. 2′, 7′-Dichlorofluorescein-diacetate (DCFH-DA) was from Applygen (C1300). Anti-ACTB (sc-47778), anti-SQSTM1 (sc-28359), and anti-CSTB antibodies (sc-6493) were from Santa Cruz Biotechnologies; anti-p-AKT (ab81283) and anti-p-mTOR (Y391) were from Abcam; TFEB (13372-1-AP) and Lamp2 (10397-1-AP) were from Proteintech; and LC3A/B (4,108) was from CST. Secondary antibodies (Alexa Fluor 488 anti-rabbit, Alexa Fluor 555 anti-rat) and Prolong anti-fade + DAPI were from Life Technologies.

### Cells, Cell Culture and Treatments

The NIH3T3 and HUVEC cell lines were obtained from Shanghai Institutes for Biological Sciences of Chinese Academy of Sciences. GFP-RFP-NIH3T3 cells are pools of NIH3T3 clones stably transfected with GFP-RFP-LC3 plasmid provided by Dr. Yoshimori (Kimura et al., 2007). Cells were cultured in Dulbecco’s modified Eagle’s medium (DMEM) supplemented with 10% fetal bovine serum (FBS) in a humidified incubator at 37°C and 5% CO_2_. The glutamine-free medium was purchased from Gibco (11960069). The method of cellular senescence induced by oxidative stress was performed according to our previous study (Han et al., 2016). For glutamine supplementation (GS), we added 20 mM glutamine to the medium and incubated the cells for 3 days based on the H_2_O_2_-induced senescence model. For glutamine deprivation (GD), cells were plated overnight in complete DMEM, briefly washed with phosphate-buffered saline (PBS) and then transferred into glutamine-free medium with 10% FBS. The corresponding glutamine-replete medium was prepared by the addition of 2 mM glutamine to glutamine-free medium. Cells were cultured for 7 days in glutamine-free medium, unless otherwise indicated.

### Fly Lifespan

The wild-type stock (Dahomey) was reared at 25°C on a 12-h on/off light cycle at 65% humidity. The females were then randomly allocated to the two groups at a density of 10 flies per vial, with 10 vials per condition (*n* = 100). The flies were transferred to a fresh food source 3 times per week, during which any deaths and censors were recorded. All lifespan experiments were repeated at least twice. Fly survival was evaluated with the log-rank test.

### Stress Assay

One hundred female flies (10 flies per vial) per treatment were fed for 10 days with a glutamine deficiency experimental diet or a control diet before being transferred to stress conditions. For the H_2_O_2_ stress assays, two groups of flies were fed medium at a concentration of 2%, and deaths were recorded every 12 h. For the starvation assay, flies were housed in vials containing 1.5% agarose with no nutritional value to provide moisture, and deaths were recorded every day. Survival for all stress assays was determined by the log-rank test.

### SA-β-Gal Staining

Intracellular and tissue senescence-associated-β-galactosidase (SA-β-gal) activity was assayed using an SA-β-gal staining kit (Beyotime, C0602) according to the manufacturer’s instructions, and senescent cells were identified as bluish green-stained cells under a phase contrast microscope. The percentage of SA-β-gal-positive cells among the total cells was determined by counting 1,000 cells in seven random fields for each group in one experiment. The results were expressed as the mean of triplicates ±SD.

### Cell Viability Assay

NIH3T3 cells were seeded at a density of 1.5 × 10^4^ ml^−1^ in 96-well plates. Twenty-4 h later, the cells were treated with glutamine-free DMEM supplemented with 10% FBS. Cell proliferation rates were subsequently assessed over time using a cell counting kit-8 (CCK-8) according to the manufacturer’s instructions.

### LC/MS/MS Measurement of Glutamine Concentration

NIH3T3 cells cultured in 100 mm dishes were treated as indicated in the figure legends. After treatment as described, the medium was discarded, and the cells were rapidly washed two times with ice-cold PBS and collected with 1 ml of PBS to prechilled 1.5 ml EP tubes. Then, the samples were centrifuged at 4°C and 1,200 rpm for 5 min, and the supernatants were discarded. The cell pellets were weighed. After total cell weight was measured, 100 µl of prechilled 60% ethanol was added to the sample for 10 min to disrupt the cells. The absolute levels of amino acids in the supernatants were quantified by amine reactive isotope-coded tags (iTRAQ Reagents; Applied Biosystems) in combination with LC/MS/MS (Chen et al., 2014).

### Real-Time Quantitative Reverse Transcription PCR

Total RNA extraction, reverse transcription and real-time PCR amplification were performed as described previously. The PCR primers for the *p16* gene were 5′-CGC​AGG​TTC​TTG​GTC​ACT​GT-3’ (forward) and 5′-TGT​TCA​CGA​AAG​CCA​GAG​CG-3’ (reverse), and those for the p53 gene were 5′-GCG​TAA​ACG​CTT​CGA​GAT​GTT-3’ (forward) and 5′-TTT​TTA​TGG​CGG​GAA​GTA​GAC​TG-3’ (reverse) for the Interleukin 8 (*il8*) gene are 5′-CGC​AGG​TTC​TTG​GTC​ACT​GT-3’ (forward) and 5′-TGT​TCA​CGA​AAG​CCA​GAG​CG-3′ (reverse), for the Autophagy related 5 (*ATG5*) gene 5′-TGT​GCT​TCG​AGA​TGT​GTG​GTT-3′ (forward) and 5′-GTC​AAA​TAG​CTG​ACT​CTT​GGC​AA-3′ (reverse) for *18S* rRNA was 5′-TTG​ACG​GAA​GGG​CAC​CAC​CAG-3’ (forward) and 5′-GCA​CCA​CCA​CCC​ACG​GAA​TCG-3’ (reverse). The experiments were performed in triplicate, and the data for the *p16, p53, IL8* and *ATG5* genes were adjusted by the values for the *18S* gene and are shown as relative fold changes compared to the control.

### Western Blot Analysis

Whole cell lysates were prepared by directly denaturing cell pellets in 1 × SDS loading buffer and then boiling for 10 min. Proteins were loaded on SDS–PAGE gels and separated by electrophoresis, followed by blotting on PVDF membranes (Millipore, Germany). The target proteins were probed with the corresponding primary antibodies under optimized conditions and then incubated with the secondary antibody. Immunological signals were surveyed *via* the electrochemical luminescence method using an Immobile Western Chemiluminescence HRP substrate kit (Millipore) and a Fusion Solo Imaging System (VIBER LOURMAT, France). Anti-p-mTOR, anti-Sequestosome-1(SQSTM1), and anti-Cathepsin B(CSTB) antibodies were used as primary antibodies. Every experiment was repeated 3 times, and representative data are shown.

### Immunofluorescence

The cells were rinsed with ice-cold PBS, fixed with 4% formaldehyde in PBS for 15 min, and permeabilized with 0.1% Triton X-100 in PBS for 15 min. After rinsing again with PBS, the cells were blocked with 0.5% BSA (Bovine Serum Albumin) for 1 h, incubated with primary antibodies in PBS with 0.5% BSA overnight at 4°C, washed three times with PBS, and incubated with fluorescent secondary antibodies in PBS overnight at 4°C. After washing with PBS, the cells were mounted on microscope slides with Prolong anti-fade + DAPI and imaged using a Leica TCS SP5-II.

### Intracellular Reactive Oxygen Species Detection

Reactive oxygen species (ROS) production was detected by the ROS detection probe DCFH-DA (Applygen, C1300) based on the mechanism by which DCFH-DA can turn to a green fluorescent molecule called DCF when oxidized by ROS so that the intracellular ROS level can be reflected by the fluorescence intensity produced by DCF. Three independent experiments were conducted.

### Animal Experiment

All procedures involving animals were performed in conformity with relevant guidelines and regulations and approved by the Ethics Committee of Sichuan University. The C57 B/L mice were purchased from the Experimental Animal Center of Sichuan University and housed five per cage under a 12/12-h dark/light cycle and standard pathogen-free conditions. During the entire experiment, food and water were available freely for these mice.

In addition, twenty-four male C57 B/L mice (12 weeks old, 18 ± 2 g) were randomly arranged into four groups evenly, including control, vehicle, D-gal and glutamine. Then, mice in the D-gal and glutamine groups experienced intraperitoneal injection every 5 days per week with D-gal (Sigma–Aldrich, MO, United States) at doses of 1,000 mg/kg for 2 months, while the mice in the vehicle group were injected with 0.9% saline for 2 months. All the mice in the glutamine group but the mice in the control, vehicle and D-Galactose (D-gal) groups were fed 3% glutamine in drinking water for 2 months. All indices were detected as described above, such as qPCR, western blot, and SA-β-gal staining.

### Superoxide Dismutase Activity Assay

Superoxide dismutase (SOD) activities were assayed using ELISA kits (A001-3; Nanjing Jiancheng Biology Engineering Institute, Nanjing, China), and absorbance was measured using an ultraviolet spectrophotometer at 450 nm.

### Statistical Analysis

Data are expressed as the means ± SD from at least three biological replicates. The difference between the control and treated groups was examined by Student’s *t* test. The difference between multiple groups was examined by one-way ANOVA with Bonferroni post hoc. *p* < 0.05 was considered to be significant, and *p* < 0.01 was considered highly significant.

## Results

### Glutamine Deprivation Induces Cellular Senescence and Aging in *Drosophila melanogaster*

**FIGURE 1:**
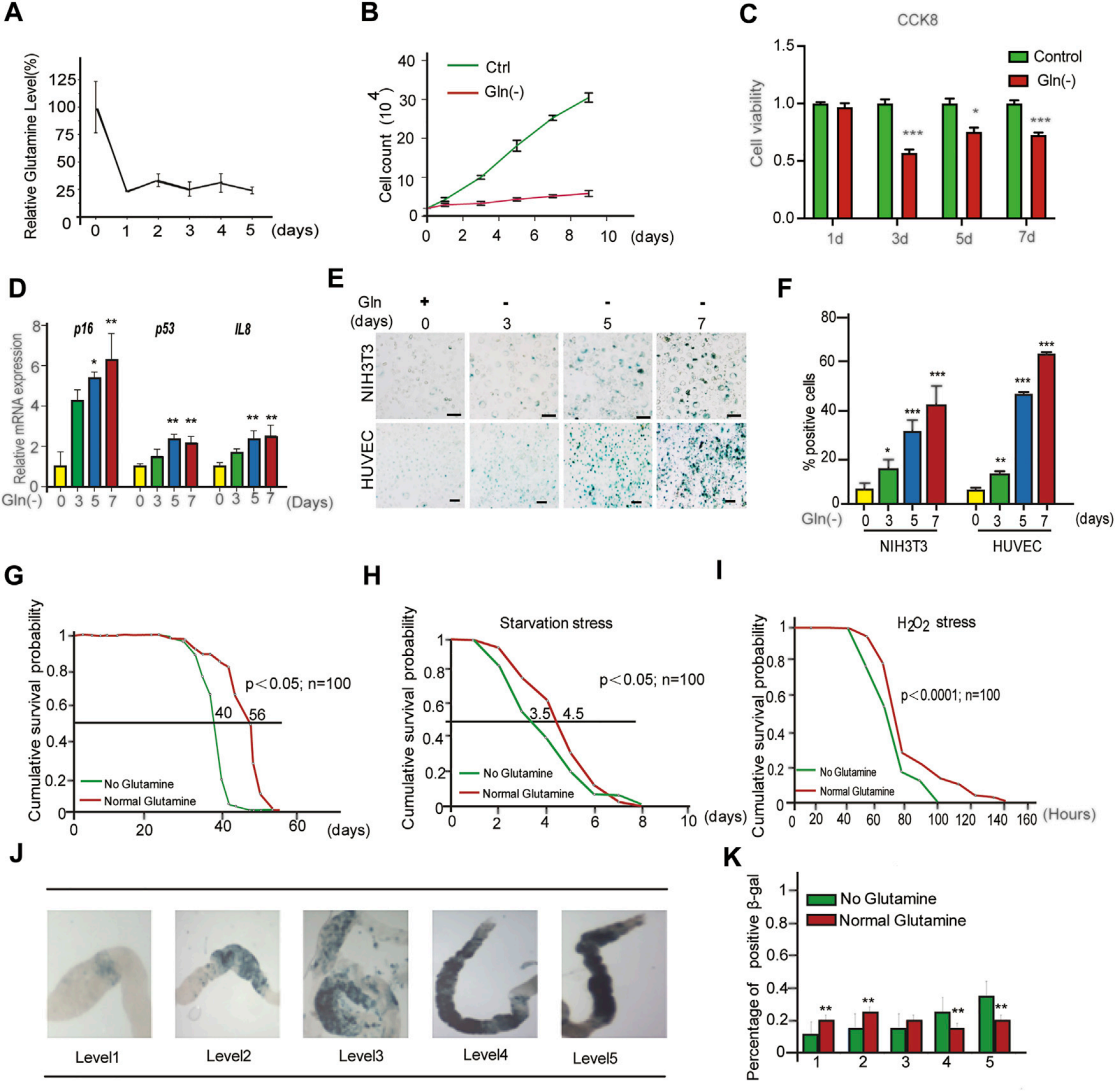
Glutamine deficiency induces cellular senescence and aging in *Drosophila melanogaster*. **(A–E)**: NIH3T3 cells were cultivated in glutamine-containing or glutamine-free medium. **(A)** Glutamine levels in cells were detected by LC/MS/MS. **(B)** Cell numbers were counted directly. **(C)** Cell viability was detected by CCK8 assay. **(D)** Relative fold-changes in the mRNA levels of the genes encoding p16, p53 and IL-8, as determined by qRT–PCR. **(E)** Images of SA-β-gal staining of NIH3T3 cells and HUVECs. **(F)** Percentages of SA-β-gal-positive cells, accounted for from images including those presented in D. **(G)** Effects of glutamine concentrations on *Drosophila* lifespan. **(H)** Starvation stress assay. (No glutamine vs. normal glutamine, *p* < 0.05; *n* = 100 flies per condition, log-rank test). **(I)** H_2_O_2_ stress assay. (No glutamine vs. normal glutamine, *p* < 0.0001, log-rank test). **(J)** SA-β-gal staining image of the intestine. **(K)** Percentages of SA-β-gal positive staining, accounted for from images including those presented in I (*n* = 100 flies). Scale bars in E = 20 μm Vs. vehicle or control: * *p* < 0.05, ** *p* < 0.01, *** *p* < 0.001.

Cellular senescence is characterized by prolonged and generally irreversible cell cycle arrest and triggers profound phenotypic changes, such as the senescence-associated secretory phenotype (SASP). First, we investigated the influence of glutamine deprivation (GD) on cellular senescence by culturing NIH3T3 cells and HUVECs in glutamine-free DMEM (supplemented with 10% FBS) for several days *in vitro*. As shown, the reduction in glutamine levels in glutamine-free DMEM-cultured cells was achieved from Day One (Figure 1A), accompanied by a significant decrease in the cell proliferation rate (Figure 1B) and cell viability (Figure 1C). However, glutamine supplementation did not rescue cell viability after glutamine deprivation (Supplementary Figure S1A). In addition, a carboxyfluorescein diacetate succinimidyl ester (CFSE) assay was adopted to monitor the number of cell divisions during proliferation (Hawkins et al., 2007). The results showed a trend toward decreased proliferation in GD cells compared to the control (Supplementary Figure S1B). From Day Five, GD induced the expression of the senescence-related genes *p16* and *p53* and the SASP gene *Il-8* (Figure 1D). Next, the mRNA levels of the SASP genes *Il-6* and *Il-1β* both increased after glutamine deprivation (Supplementary Figures S1C,D). A high rate of SA-β-gal-positive cells was also observed in both the NIH3T3 and HUVEC populations (Figures 1E,F). Similar evidence was obtained after treatment with DON, a chemical inhibitor of glutaminase (GLS) that catalyzes the first step of glutamine catabolism (Supplementary Figure S1E). These results reveal that GD reduces intracellular glutamine levels and then facilitates cellular senescence.

To obtain evidence *in vivo*, a GD diet was carried out in *Drosophila melanogaster*. First, the lifespan of flies fed GD-free fodder was shorter than that of flies fed normal fodder (Figure 1G). Similar to GD, DON also shortened the lifespan of flies (Supplementary Figure S1F). Next, the healthy lifespan of flies was evaluated by the starvation resistance assay. GD-fed flies showed lower resistance to starvation (Figure 1H). Third, GD-fed flies became much weaker when encountering a toxic dose of H_2_O_2_ (Figure 1I). Finally, SA-β-gal staining of the gut in GD-fed flies was markedly increased (Figures 1J,K). Together, these data suggest that GD can induce senescence in cells and promote aging in flies.

### Glutamine Deprivation Sustainably Activates the Akt-mTOR Pathway

**FIGURE 2:**
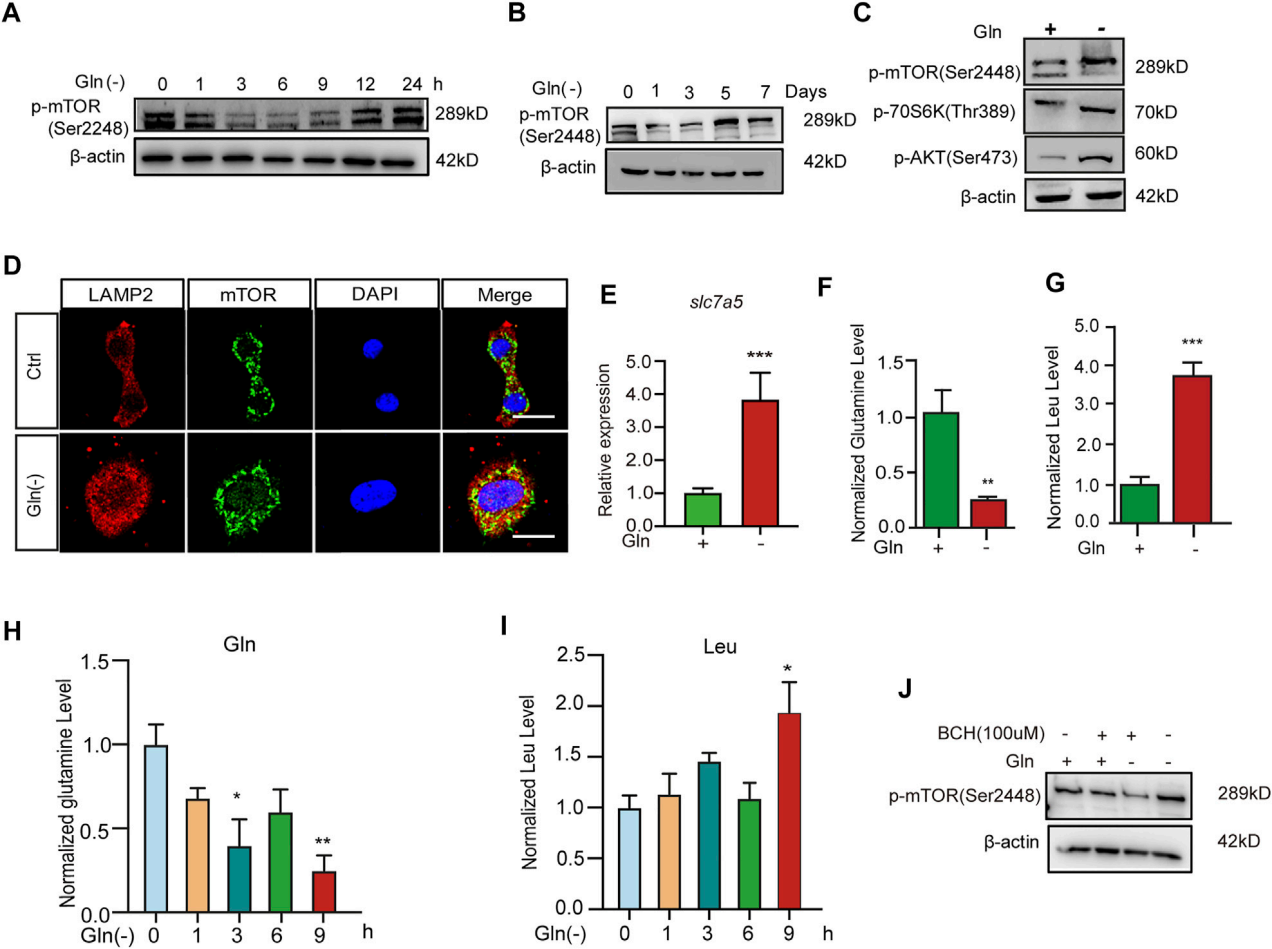
Glutamine deprivation causes sustained Akt-mTOR pathway activation. NIH3T3 cells were incubated in glutamine-free [Gln (-)], complete (Ctrl) DMEM as indicated. **(A,B)** Images of immunoblots against p-mTOR and β-actin. **(C)** Images of immunoblots against p-mTOR, p70s6k, p-AKT and β-actin. **(D)** Immunofluorescent images of mTOR and Lamp2 in cells. **(E)** Relative fold-changes in the mRNA level of the slc7a5 gene were detected by qRT–PCR. **(F–I)** The levels of glutamine and leucine in NIH3T3 cells by LC/MS/MS. **(J)** Images of immunoblots against p-mTOR and β-actin. Scale bars in D = 20 μm Vs. vehicle or control: * *p* < 0.05, ** *p* < 0.01, *** *p* < 0.001.

To explore the mechanism of cellular senescence and aging induced by long-term GD, we investigated its impact on the mTOR cascade. Our results showed that GD decreased the phosphorylation of mTOR in the short term for less than 12 h (Figure 2A). However, the phosphorylation of mTOR (Figure 2B), Akt (Figure 2C) and S6K (Figure 2C) increased after more than 5 days of glutamine deprivation. Furthermore, the colocalization of mTOR protein with lysosomal membrane protein Lamp2 also increased after long-term GD (Figure 2D). Solute carrier family seven member 5 (SLC7A5) is a leucine transporter that stimulates mTOR activation (Sokolov et al., 2020). Our results showed that the expression of SLC7A5 increased after long-term GD (Figure 2E). Based on previous reports that mTOR is a leucine sensor, the intracellular levels of glutamine and leucine were quantified in parallel by LC–MS/MS assay. The results showed that the level of glutamine was decreased and leucine was increased after long-term GD (Figures 2F,G), which was consistent with the previous demonstration that leucine availability upregulates mTOR activity (Son et al., 2020). Similar to the level of leucine in long-term GD, the level of glutamine was decreased and leucine was increased after short-term GD (Figures 2H,I). Finally, 2-aminobicyclo-(2,2,1)-heptane-2-carboxylic acid (BCH), an inhibitor of SLC7A5, was used to inhibit L-leucine transport. Our results showed that the phosphorylation of mTOR decreased after BCH treatment compared to glutamine deprivation treatment alone (Figure 2J). These results indicate that GD induces sustained mTOR activation.

### Glutamine Deprivation Impairs Autophagic Flux and Lysosomal Function

**FIGURE 3:**
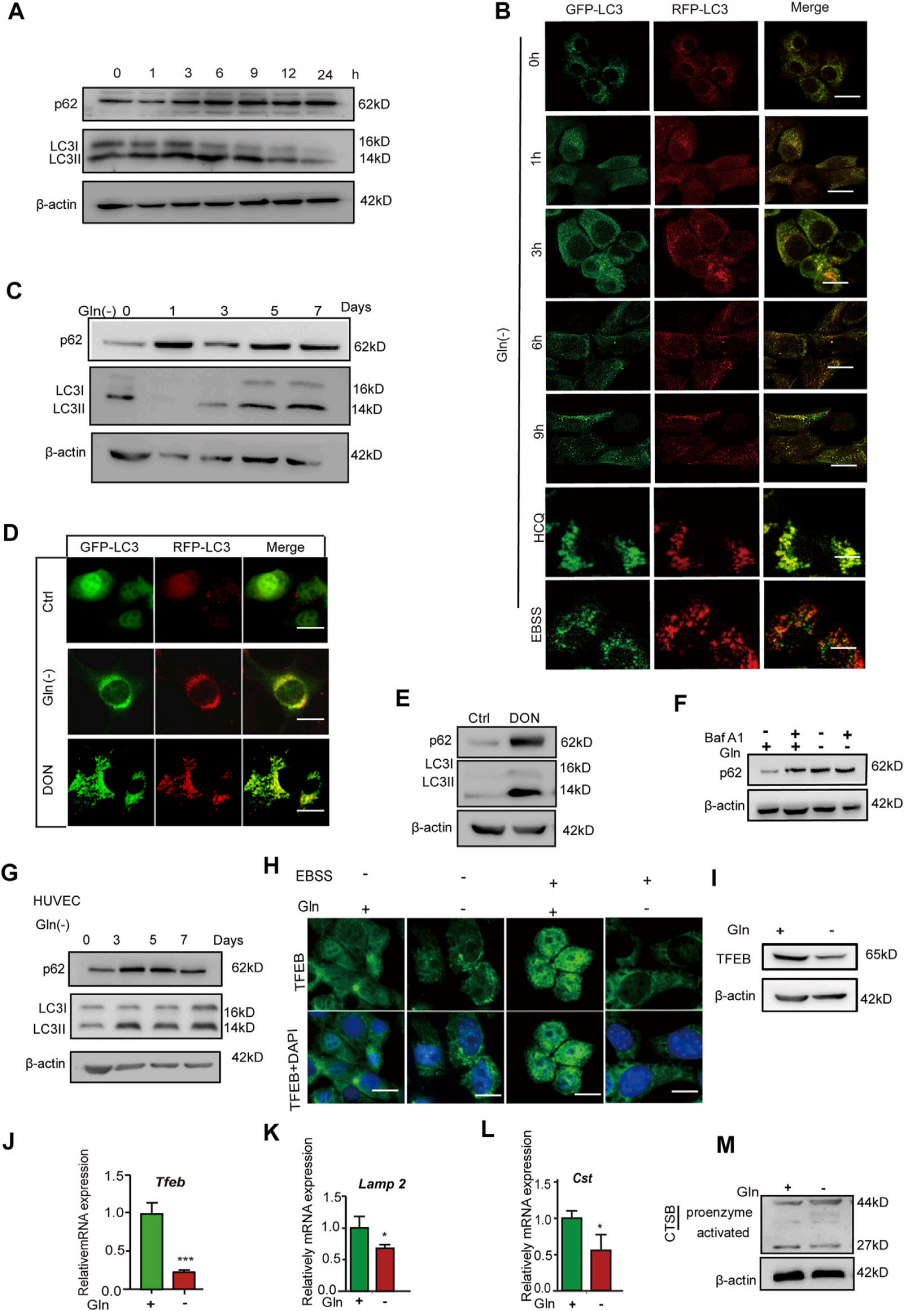
Glutamine deficiency induces autophagy impairment and lysosome dysfunction. **(A)** NIH3T3 cells were incubated for the indicated hours, and images from immunoblot assays against p62 and β-actin are shown. **(B)** GFP-RFP-LC3-expressing NIH3T3 cells incubated in complete (Ctrl) or glutamine-free [Gln (-)] DMEM for 9 h or treated with HCQ (10 nM) or EBSS for 12 h. Fluorescence images of GFP, RFP and the overlap are shown. **(C)** NIH3T3 cells were incubated for the indicated days, and images from immunoblot assays against p62 and β-actin are shown. **(D)** GFP-RFP-LC3-expressing NIH3T3 cells incubated in complete (Ctrl) or glutamine-free (Gln (-)) DMEM for 7 days or treated with DON (10 nM) for 5 days. Fluorescence images of GFP, RFP and the overlap are shown. **(E)** NIH3T3 cells were incubated with DON for 5 days, and images from immunoblot assays against p62 and β-actin are shown. **(F)** NIH3T3 cells incubated for 24 h, with or without the addition of bafilomycin A1 (Baf A1, 25 μM), and images from immunoblot assays against p62 and β-actin are shown. **(G)** HUVECs were incubated with glutamine-free [Gln (-)[ DMEM for 3–7 days. **(H)** Immunofluorescence images of TFEB in cells treated with EBSS for the last 24 h in the indicated groups. **(I)** Images of immunoblots against TFEB and β-actin. **(J–L)** Relative fold-changes in the mRNA levels of genes encoding TFEB, Lamp1 and CTSB, as determined by qRT–PCR. **(M)** Images of immunoblots against cathepsin B and β-actin. Vs. vehicle or control: * *p* < 0.05, ** *p* < 0.01, *** *p* < 0.001. Scale bars = 10 μm.

mTOR signaling has been regarded as a negative regulator of autophagy, and autophagy impairment is an important characteristic of cellular senescence (Tai et al., 2017; Jia et al., 2019; Nowosad et al., 2020); thus, we investigated the autophagy status in GD cells. As shown in Figure 3A, the level of p62 protein and the ratio of LC3II/I both increased after short-term GD (within 9 h). NIH3T3 cells stably expressing mRFP‐GFP‐LC3 were employed to visualize yellow (RFP + GFP+) and red (RFP + GFP-) LC3 puncta, which represent blocked and activated autophagic flux, respectively (Klionsky et al., 2016). In this assay, GFP fluorescence (green) is rapidly quenched in the acidic environment, while RFP fluorescence (red) remains stable and serves as a more specific marker of LC3B expressed in autolysosomes. When autophagy flux was activated, the LC3 puncta are shown in red. When autophagic flux was blocked, the LC3 puncta appeared yellow (Tai et al., 2017). The Figure 3B shows that the LC3 puncta tended to become yellow after short-term GD. Furthermore, we investigated autophagic flux under long-term glutamine deprivation. We found that p62 protein dramatically accumulated and that the level of LC3II/LCI increased after treatment with long-term GD (Figure 3C), as well as in DON-treated cells (Figure 3E). The LC3 puncta also turned yellow in long-term GD cells (Figure 3D). Bafilomycin A1 is reported to be an autophagic inhibitor that disrupts autophagic flux by independently inhibiting V-ATPase-dependent acidification and Ca-P60A/SERCA-dependent autophagosome-lysosome fusion (Mauvezin and Neufeld, 2015). However, bafilomycin A1 treatment did not induce further aggregation of the p62 protein in long-term GD cells, which indicated that GD-induced p62 accumulation resulted from impaired autophagy rather than elevated p62 induction (Figure 3F). Consistent results were also obtained in HUVECs (Figure 3G). Finally, we measured lysosome function owing to its crucial role in the late-stage flow of autophagy. As shown, the expression of TFEB, a prime transcription factor for the expression of a series of autolysosomal genes, was repressed in GD cells (Figures 3H–J), as well as the target genes TFEB*, Lamp1, and Ctsb* (Figures 3K,L). Moreover, the level of activated cathepsin B protein was reduced in long-term GD cells (Figure 3M). Collectively, these results reveal that long-term GD induced autophagy flux impairment and lysosome dysfunction.

### Akt-mTOR Inactivation Restores GD-Induced Autophagy Impairment and Cellular Senescence

**FIGURE 4:**
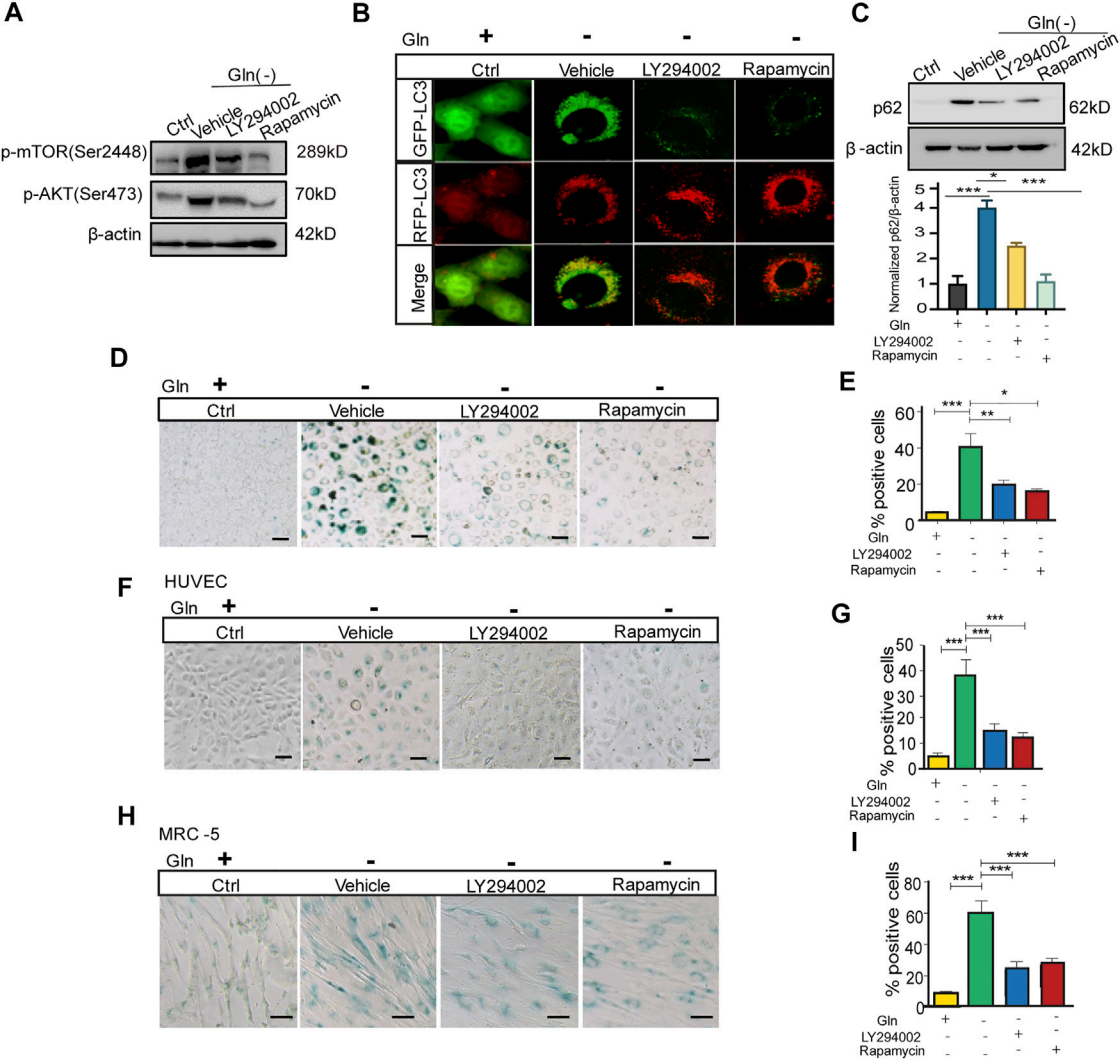
mTOR inactivation ameliorates autophagy impairment and senescence caused by glutamine deprivation. NIH3T3 cells, mRFP-GFP-LC3 NIH3T3 cells, HUVECs or MRC-5 cells were treated with glutamine-free DMEM for 7 days and treated with 10 µM LY294002 (PI3k inhibitor) or 100 nM rapamycin (mTOR inhibitor) for 24 h. **(A)** The images from immunoblot assays against p-AKT, p-mTOR and β-actin are shown in NIH3T3 cells. **(B)** Fluorescence images of GFP and RFP and the overlap are shown in mRFP-GFP-LC3 NIH3T3 cells. **(C)** The images from immunoblot assays against p62 and β-actin are shown. **(D)** Images of SA-β-gal staining of NIH3T3 cells are shown. **(E)** Percentages of SA-β-gal-positive cells, accounted for from images including those presented in D. **(F)** Images of SA-β-gal staining of HUVECs are shown. **(G)** Percentages of SA-β-gal-positive cells, accounted for from images including those presented in F. **(H)** Images of SA-β-gal staining of MRC-5 cells are shown. **(I)** Percentages of SA-β-gal-positive cells, accounted for from images including those presented in H. Scale bars in B = 10 μm. Scale bars in D, F and H = 20 μm Vs. vehicle or control: * *p* < 0.05, ** *p* < 0.01, *** *p* < 0.001.

Then, we checked whether inactivation of mTOR could rescue autophagy impairment and senescence in GD cells. GD cells treated with the PI3K/Akt inhibitor LY294002 and the mTORC1 inhibitor rapamycin had lower levels of Akt and mTOR phosphorylation than untreated cells (Figure 4A). Coincidentally, LC3 puncta in GD cells tended to become red (GFP-/RFP+) upon treatment with these inhibitors (Figure 4B), together with a decreased protein abundance of p62 (Figure 4C). Importantly, Figure 4C treatment with LY294002 or rapamycin also attenuated senescence induced by GD, represented by weaker SA-β-gal positive staining (Figure 4D) and a decreased proportion of SA-β-gal positive cells (Figure 4E). Consistent results were collected in HUVECs (Figures 4F,G) and MRC-5 cells (Figures 4H,I). These results suggest that blocking the Akt-mTOR signaling pathway can effectively mitigate autophagy impairment and cellular senescence induced by GD.

### Glutamine Supplementation Alleviates Cellular Senescence and the Aging Phenotype in Mice Induced by Oxidative Stress

**FIGURE 5:**
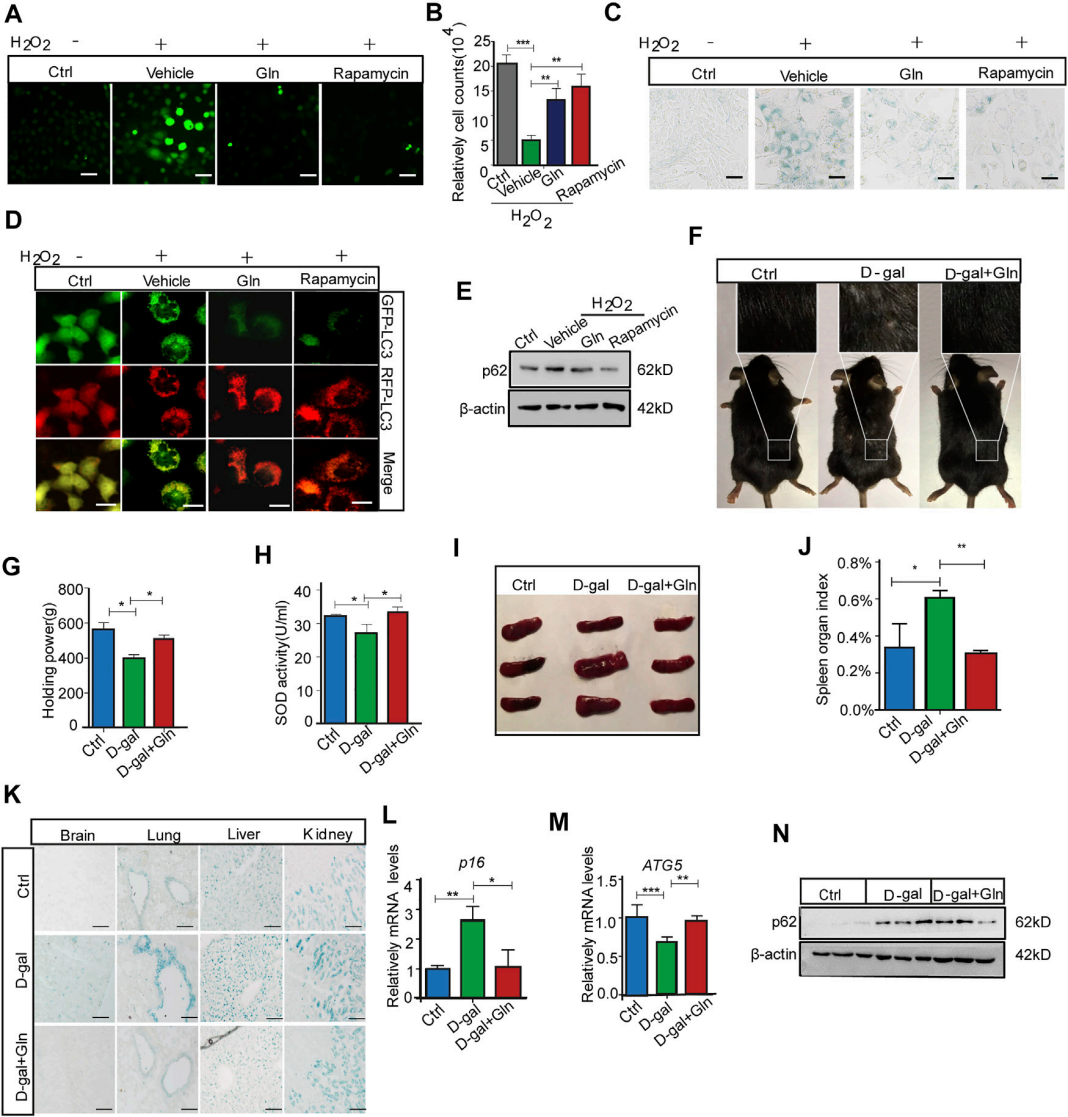
Glutamine supplementation (GS) rescued oxidative stress-induced cellular senescence and aging. NIH3T3 cells were treated with PBS (Ctrl) or 400 μM H_2_O_2_ in PBS for 45 min and then cultured in complete medium for 3 days with 20 mm glutamine or 100 nm rapamycin (positive control). **(A)** The level of ROS indicated by DCFH-DA fluorescence in NIH3T3 cells induced by H_2_O_2_. **(B)** Cell numbers were counted directly. **(C)** Images of SA-β-gal staining of NIH3T3 cells. **(D)** Fluorescence images of GFP and RFP and the overlap are shown in mRFP-GFP-LC3 NIH3T3 cells. **(E)** The images from immunoblot assays against p62 and β-actin are shown. C57BL/6 adult mice were given a peritoneal injection with or without 1,000 mg/kg/d D-gal, while D-gal-injected mice were supplied with or without 3% glutamine in drinking water for over 2 months. **(F)** The image of hair luster and volume descendant. **(G)** The grasping force of mice. **(H)** Serum SOD activity in mice. **(I)** Image of the spleen in mice. **(J)** The spleen index was determined. **(K)** Images of SA-β-gal staining in the brain, liver, lung and kidney tissues of mice. **(L)** Relative fold-changes in the mRNA levels of the genes encoding ATG5 in brain tissue, as determined by qRT–PCR. **(M)** Relative fold-changes in the mRNA levels of the genes encoding p16 in brain tissue, as determined by qRT–PCR. **(N)** Images of immunoblot assays against p62 and β-actin. Each experiment was tested with over 12 mice and repeated 3 times. Scale bars in A and C = 10 μm. Scale bars in D = 20 μm. Scale bars in K = 50 μm Vs. vehicle or control: * *p* < 0.05, ** *p* < 0.01, *** *p* < 0.001.

We further collected evidence that glutamine supplementation (GS) could alleviate senescence, especially the premature senescence induced by oxidative stress. The test was conducted by loading additional glutamine in H_2_O_2_-treated fibroblast cells, where H_2_O_2_ treatment worked as an oxidative stress inducer to evoke premature senescence (Zhou et al., 2014). The results showed that GS not only decreased the ROS level marked by DCFH-DA fluorescence (Figure 5A) but also facilitated cell proliferation similar to rapamycin (positive control) (Figure 5B). In addition, GS treatment induced a decrease in SA-β-gal staining in H_2_O_2_-treated cells (Figure 5C). Consistently, with GS treatment, cells stably expressing the mRFP-GFP-LC3 fusion protein showed fewer red (RFP-GFP+) LC3 puncta (Figure 5D) and decreased levels of p62 protein (Figure 5E). In addition, GS treatment did not decrease SA-β-gal staining in H_2_O_2_-treated atg7 knockout cells compared to that in wild-type cells (Supplementary Figure S2). To further verify the suppressive effect of GS on aging *in vivo*, we performed GS experiments in a D-galactose (D-gal)-induced progeria mouse model. Several animal models have been proposed to investigate the mechanisms of aging. The D-galactose (D-gal) model is considered one of the more affordable progeria mouse models because of its few side effects and high survival rate (Aydin et al., 2012). Thus, it is suitable for antiaging studies. The results showed that the gloss and density of hair decreased obviously in D-gal-treated model mice, while the appearance improved remarkably in mice treated with GS (Figure 5F). Moreover, the muscle tension of GS-treated mice was significantly restored (Figure 5G), and the SOD activity in the serum of these mice also increased markedly (Figure 5H). Supporting data obtained from the spleen index measurement showed that the index was increased in the D-gal group but returned to normal in the GS group (Figures 5I,J). Consistently, the SA-β-gal positive staining in brain, lung, liver and kidney tissues all decreased obviously in the GS group (Figure 5K). Furthermore, the expression of *p16* (Figure 5L) in brain tissue was also decreased in GS mice compared to D-gal mice. Additionally, autophagy activity was also determined to confirm improved autophagy, with increased expression of *Atg5* (Figure 5M) and increased expression of p62 protein (Figure 5N). Collectively, these results reveal that GS can effectively prevent oxidative stress-induced senescence and aging, together with improved autophagy activity.

## Discussion

In this research, we revealed that long-term glutamine deprivation (GD) can induce cellular senescence and aging in *Drosophila melanogaster* and that glutamine supplementation (GS) can ameliorate the cellular senescence caused by H_2_O_2_ and the aging phenotypes of mice induced by D-gal. Our results also confirmed that sustained mTOR activation and resultant autophagy impairment are involved in the glutamine availability-regulated aging process. These findings provide a new mechanistic explanation for the importance of glutamine availability and suggest that glutamine may be a potential antiaging nutrient.

Glutamine plays an essential role in an organism’s internal environment. Changes in its concentration have a remarkable effect on the function of the majority of organ systems, such as the brain (Baek et al., 2020), muscles (Shang et al., 2020) and heart (Salabei et al., 2015). It has been reported that GS is meaningful for improving the inflammatory status and redox balance in the elderly population (Cruzat et al., 2018; Almeida et al., 2020; Amirato et al., 2021), while GD can cause proliferative inhibition, ROS production and cell cycle disruption (Gwangwa et al., 2019). Indeed, our results showed that glutamine deprivation could induce ROS production. This may be due to the impairment of mitochondrial function caused by glutamine deprivation (Supplementary Figure S3A). However, N-acetylcysteine (NAC), as an antioxidant, reduced ROS and SA-β-gal staining caused by glutamine deprivation (Supplementary Figures S3B,C). These results suggest that glutamine deficiency may also cause a disturbance of the redox state, thereby accelerating cellular senescence. The following GD and GS experiments *in vitro* and *in vivo* demonstrated that glutamine availability is important for redox maintenance and aging protection. However, the mechanism needs to be further investigated.

The role of autophagy in aging has attracted increasing attention. Recently, autophagy impairment has been regarded as a feature of senescent cells, and it is clear that autophagy activation can resist cellular senescence (Garcia-Prat et al., 2016; Tai et al., 2017; Ma et al., 2018). However, there is currently controversy regarding the relationship between autophagy function and glutamine availability. For example, Song Zhao and Christina H Eng et al. (2010) found that either glutamine or its catabolic product ammonia could induce autophagy in cancer cells (Zhao et al., 2019). Kristan E. van der Vos et al. (2012) further demonstrated that glutamine metabolism can directly activate autophagy *via* the PI3K–PKB–FOXO network. The findings in our study are consistent with this concept, although our evidence was collected mainly from glutamine deprivation. Conversely, we also note the study of Yuhua Zhu et al. (2015) which reported that GD activates autophagy in porcine cells. However, our findings showed that glutamine deprivation resulted in autophagy inhibition. Therefore, the implication of glutamine availability on autophagy cannot be summarized consistently, which may be the matter of the difference in cell function and cell metabolism. Specifically, this is due to differences in cellular demand for glutamine and differences in basal autophagic activity. Actually, the diversities mainly come from the culture conditions. We noticed that in the study by Yuhua Zhu et al. (2015) cells were cultured in serum- and Gln-free DMEM, whereas we used glutamine-free DMEM supplemented with FBS. Given that FBS contains multiple components that affect cell metabolism and stress responses, it may not be inconceivable that the two conditions produce different outcomes. Therefore, the effect of glutamine availability on autophagy regulation and its mechanism are important issues that need further explanation and investigation.

Although the precise mechanisms involved in glutamine-mediated regulation of autophagy remain elusive, the mTOR signaling pathway may be a clue to study. This is because of the important role of mTOR in cell survival and proliferation and the association between glutamine and mTOR pathways (Ravikumar et al., 2004; Jung et al., 2009; Kim and Guan, 2015). In previous studies, glutamine has been implicated in the activation of mTORC1 to support rapid cell proliferation (Feng et al., 2018; Shang et al., 2020). However, in our study, glutamine was a negative regulator of mTOR activity with the persistent activation of mTORC1 in glutamine-deprived cells. It is worth noting that the cellular leucine level was found to increase after long-term glutamine deprivation. This is one of the possible explanations for GD-induced mTOR activation. Leucine has been reported to be a potent stimulator of mTORC1: it blocks the inhibitory effect of the protein sestrin two on the GATOR2 complex that activates mTORC1 (Wolfson et al., 2016). In addition, we also found that SLC7A5 expression was increased in GD cells. SLC7A5, which is a bidirectional transporter on the plasma membrane, promotes the influx of leucine/EAAs and the efflux of glutamine simultaneously (Nicklin et al., 2009). Research from Viktor I. Korolchuk et al. (2011) suggested that the position of lysosomes coordinates with mTORC1 activity in response to nutrient availability, especially amino acids (Carroll et al., 2017). Additionally, Rag-Ragulator-mediated translocation of mTORC1 to lysosomal membranes is essential for mTORC1 activation (Sancak et al., 2010). Another explanation for mTOR activation in our study is the translocation of mTOR complex one to lysosomes (Jewell et al., 2015), as supported by our data in Figure 2D. Conversely, GD-induced mTOR inactivation occurred within 12 h (Chen et al., 2014). In a time-course survey, mTOR activity exhibited a two-stage alteration, reducing within 9 h but elevating from day 5 under glutamine deprivation conditions (Figure 2B). This interesting phenomenon indicated that mTOR activity can fluctuate with the time of glutamine deprivation, down quickly and then up after several days. The fluctuation of mTOR activity suggests that the implication of glutamine availability on mTOR activity is a delicate and complicated issue, also means it deeply takes part in the precise regulation of mTOR activity and downstream autophagy, as well as aging process.

## Conclusion

This study verified that long-term GD induces aging *in vitro* and *in vivo*, while GS rescues the aging induced by oxidative stress. Importantly, this study demonstrates that long-term GD could activate the mTOR-TFEB axis to inhibit autophagic flux, suggesting that glutamine availability participates in the regulatory mechanism upon aging development. It also confirms the biological role of glutamine and indicates its potential for further medical application. Certainly, deep-going studies are needed to obtain more insights into the advanced mechanism of glutamine on aging and autophagy.

## Data Availability

The original contributions presented in the study are included in the article/Supplementary Material, further inquiries can be directed to the corresponding author.
