# Supplementary material for: Glutamine Availability Regulates the Development of Aging Mediated by mTOR Signaling and Autophagy
Source: Front Pharmacol. 2022 Jul 4;13:924081. doi: 10.3389/fphar.2022.924081 (PMC9289448; doi:10.3389/fphar.2022.924081)

**Supplementary Figure 1**


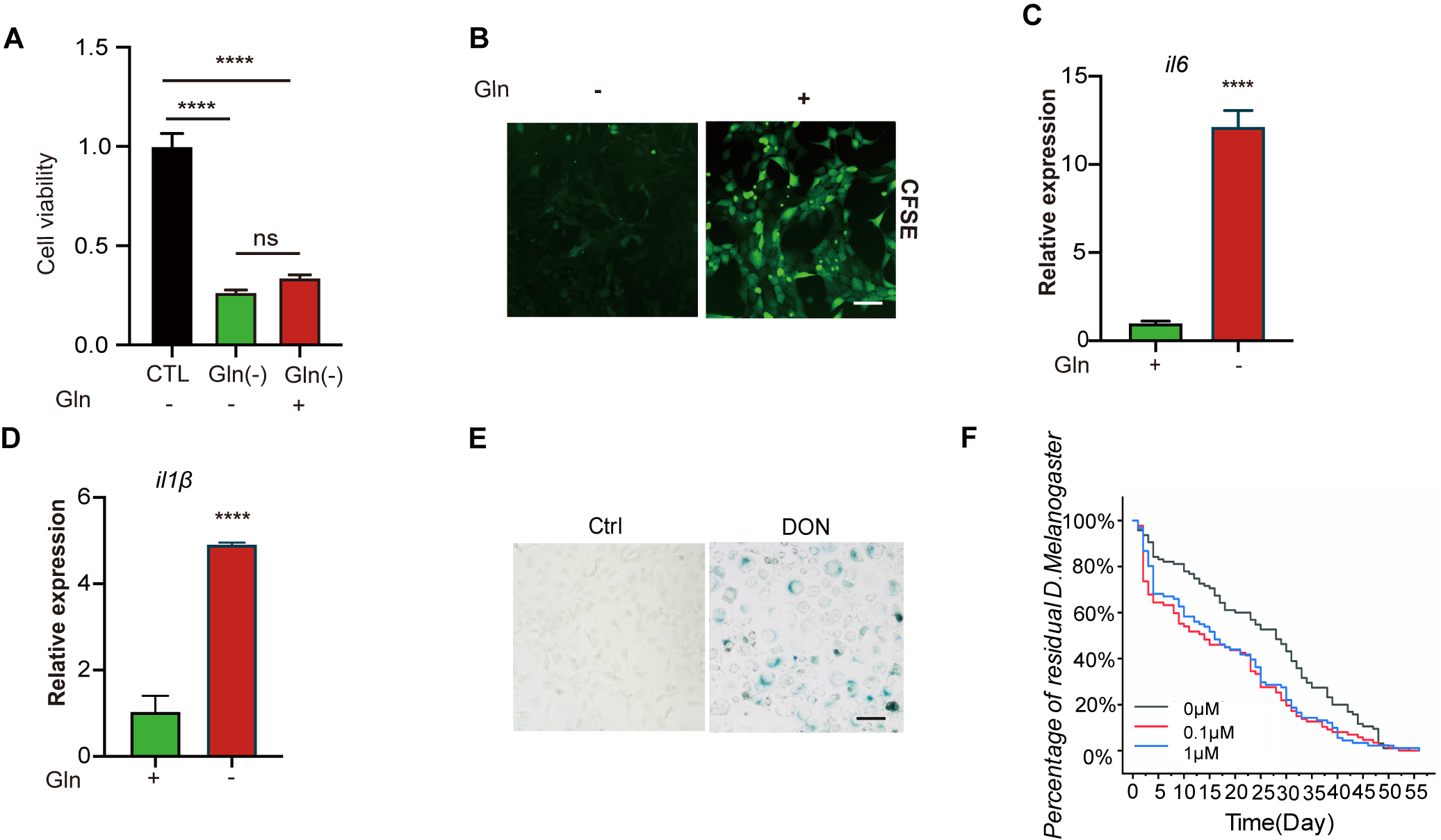


**Supplementary Fig1.Glutamine deficiency could** [**accelerate**](javascript:;) **the cellular senescence and aging of *Drosophila melanogaster*.** (A) NIH3T3 cells were cultured with glutamine deprivation for 7 days and then incubated with glutamine for 24 h. CCK8 was used to detected the cellular viability. (B) Cells were labeled with CFSE at 10 μM /L and treated with Gln-free DMEM for 7 days. The images of CFSE were captured by fluorescence microscope. (C-D) Relative fold-changes in mRNA level of genes encoding IL-6 and IL-1β, determined by qRT-PCR. (E)The image of SA-β-gal staining in NIH3T3 cells treatment with DON (10nM) for 5 days. (F)Treatments with different concentration glutamine inhibitor (DON) shorten the lifespan in 30-day old flies. Scale bars in B and E= 20μm. (Vs vehicle or control: * P < 0.05, ** P< 0.01, *** P<0. 001, ****P < 0.0001, n=100 flies per condition, log-rank test).

**Supplementary Figure 2**

**Supplementary Fig2. The anti-aging effects of glutamine depends on autophagy. The** wildtype NIH3T3 and ATG7 KO NIH3T3 cells were treated with PBS（Ctrl） or with 400 μM H_2_O_2_ in PBS for 45 minutes, and then cultured in a complete medium for 3 days with 20mm glutamine. **(A)** Images of SA-β-gal staining of wildtype NIH3T3 and ATG7 KO NIH3T3 cells. Scale bars = 20μm.

**Supplementary Figure 3**


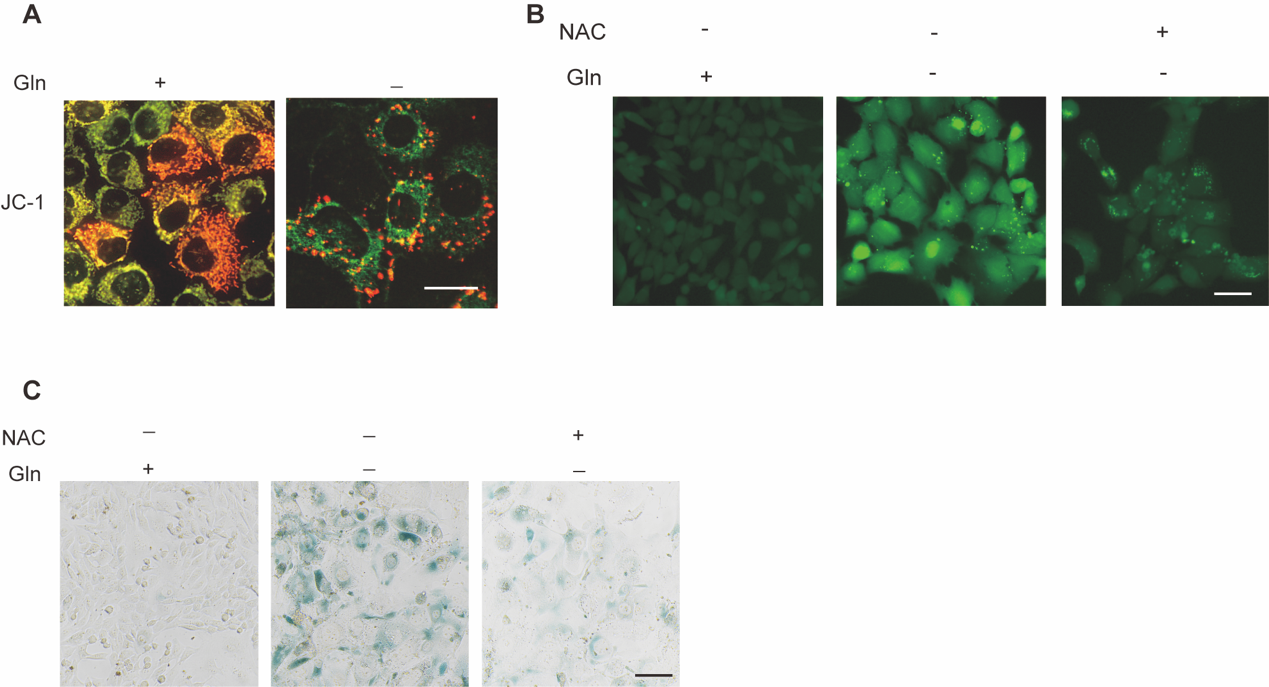


**Supplementary Fig3. Glutamine deficiency impairs mitochondrial function and redox state imbalance. (A)** NIH3T3 cells were treated with glutamine deprivation for 7 days. Images of JC-1 staining were captured. NIH3T3 cells were cultured with glutamine deprivation for 7 days and then incubated with NAC (2μM) for 24 h. (B) The level of ROS indicated by DCFH-DA fluorescence in NIH3T3 cells. (C) Images of SA-β-gal staining of NIH3T3 cells. Scale bars = 10μm.

**Supplementary Figure 4**

Figure2


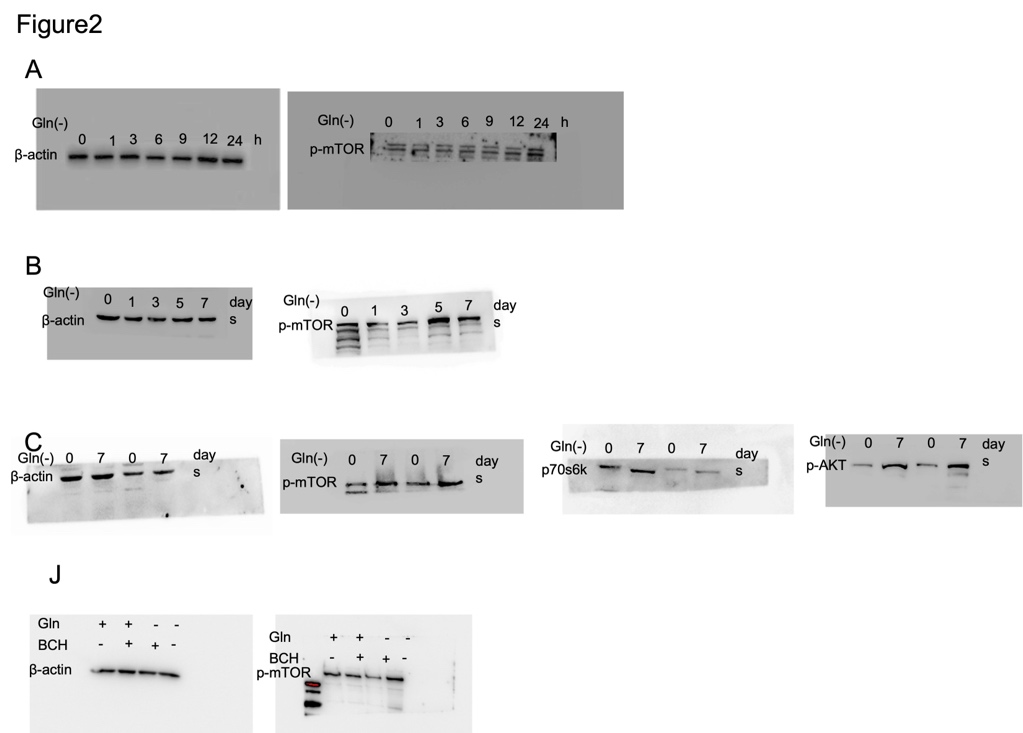


Figure3


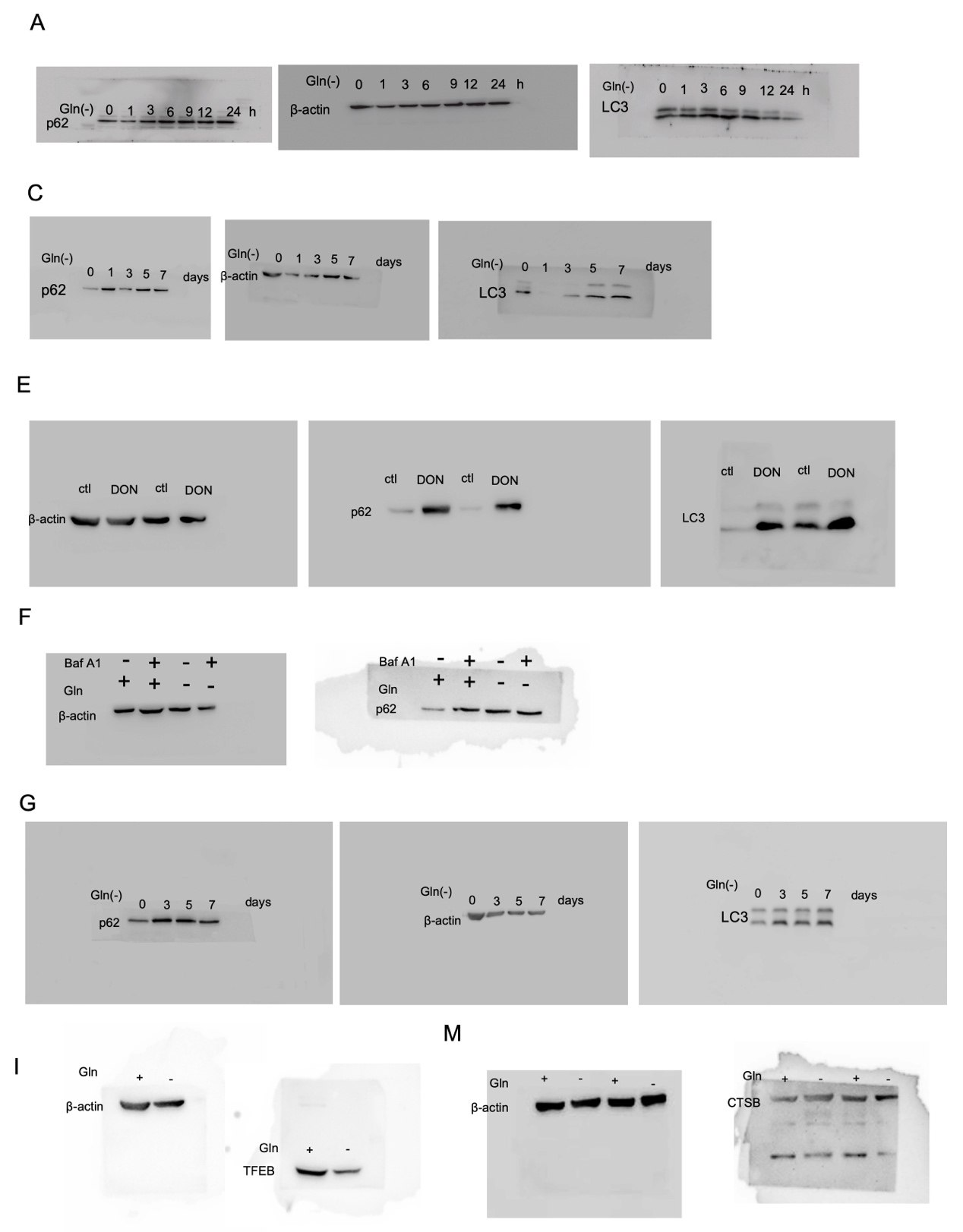


Figure4


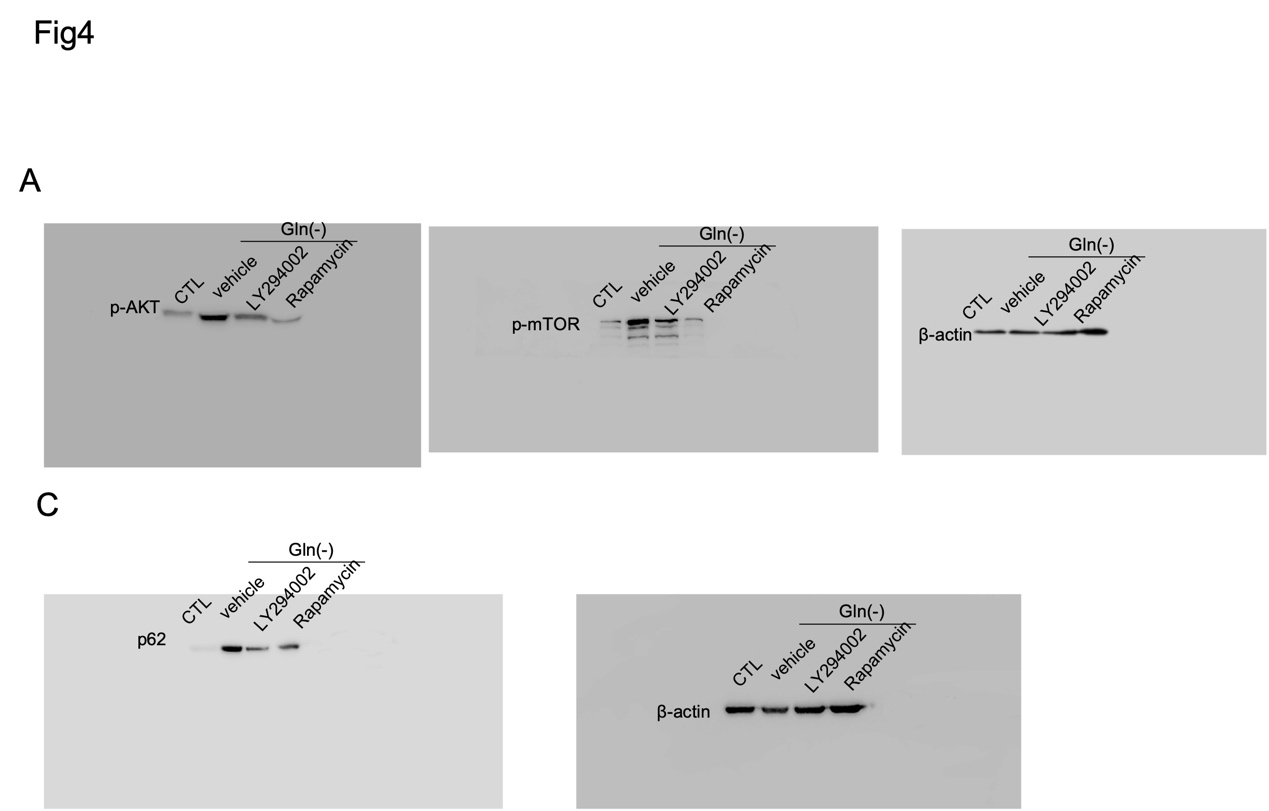


Figure5
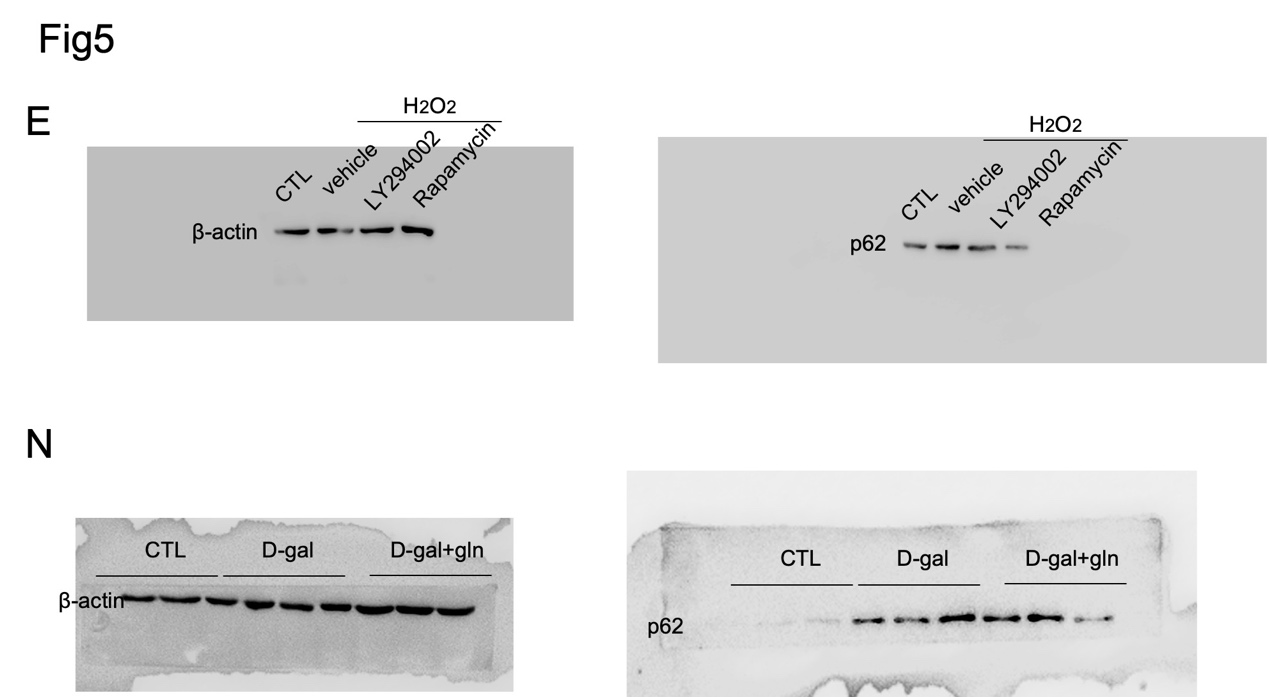

Supplement: Supplementary file 1 [file DataSheet1.docx]
